# Individual Patient Sociodemographic Characteristics Are Associated With Waiting Time to Physician Assessment in Seven Swedish Emergency Departments: An Observational 5-Year Cohort Study of Emergency Department Visits in the Stockholm Region

**DOI:** 10.1016/j.acepjo.2025.100250

**Published:** 2025-09-30

**Authors:** Gustav Malmer, Anna Fällman, Richard Åhlberg, Per Svensson, Eli Westerlund, Björn af Ugglas

**Affiliations:** 1 Department of Clinical Sciences, Danderyd Hospital, Karolinska Institutet, Stockholm, Sweden; 2 Department of Emergency Medicine, Karolinska University Hospital, Stockholm, Sweden; 3 Department of Clinical Science and Education, Södersjukhuset, Karolinska Institutet, Stockholm, Sweden; 4 Department of Learning, Informatics, Management and Ethics, Karolinska Institutet, Stockholm, Sweden

**Keywords:** emergency department, Sex, socioeconomic status, patient age, educational level, place of birth, waiting time to physician, equity, inequity

## Abstract

**Objectives:**

Emergency department (ED) inequities in access, treatment, and flow have been reported, but equity in the ED remains understudied. This study aimed to determine the association between patients’ sociodemographic characteristics and waiting times to physician assessment in the ED.

**Methods:**

This was an observational study on 1,744,301 ED visits by 884,228 patients, using registry data from all 7 EDs in the Stockholm region, Sweden, 2012 to 2016. Exposures were age, sex, educational level, and birth region. A multivariable generalized linear regression model with penalized quasi-likelihood, adjusted for triage priority, chief complaint, admission status, crowding level, shift, weekend, and hospital site, was used to analyze the associations.

**Results:**

In the adjusted model, women waited 1% longer than men (95% CI, 1%-2%). Patients aged 60 to 79 years waited 8% longer than those aged 18 to 39 years (95% CI, 7%-8%). Those born outside the EU or Nordic countries waited 6% longer than Swedish-born patients (95% CI, 6%-6%). Patients with less than 10 years of education waited 2% longer than those with over 12 years (95% CI, 1%-2%).

**Conclusion:**

Waiting time to a physician was independently associated with age, sex, education, and birth region. Individuals who were older than 60 years and born outside Europe were the strongest predictors of longer waits. These findings suggest disparities not explained by medical need. As longer waits are linked to worse outcomes, further research is needed to understand mechanisms and reduce inequities in emergency care.

**The Bottom Line**

We studied whether age, sex, education, and birth region are linked to time to physician assessment in emergency departments. In 1.7 million visits, all factors were significant, after adjusting for clinical factors and the emergency department (ED) context. Those over 60 years and being born outside Europe were the most powerful predictors. Women over 80 years, born outside Europe, with less than 10 years of education, waited 14 minutes longer than young, highly educated Swedish-born men. These findings suggest that the ED system, processes, and working methods may result in inequities. Further investigation is needed, as longer waits can increase the risk of poor outcomes.

## 1 Introduction

### 1.1 Background

Inequities in the emergency department (ED) care persist across all stages: access, triage, assessment, treatment, and boarding. These patterns may be shaped by systemic bias, both explicit and unconscious.^1^, ^2^, ^3^ Waiting time to the physician is an essential aspect of the ED visit, associated with patient experience and adverse outcomes.^4^, ^5^, ^6^, ^7^, ^8^

### 1.2 Importance

International studies have shown that marginalized groups—such as ethnic minorities and those with lower socioeconomic status—often wait longer and receive lower quality care.^3^^,^^9^, ^10^, ^11^, ^12^ This has also been shown in a Swedish setting for chest pain patients specifically.^13^

Research on age and sex inequities in the ED remains limited.^3^^,^^9^, ^10^, ^11^^,^^13^ Older patients, who often present with frailty and a higher risk of adverse outcomes, may be especially vulnerable to delayed care.^14^^,^^15^ Two European single-center studies have reported longer physician waiting times for older patients, but adjusted only for triage priority or time of presentation.^16^^,^^17^

To our knowledge, no previous studies have examined equity in a Scandinavian unsorted general ED population, while adjusting for both clinical factors and ED context.

### 1.3 Goals of this investigation

The objective of this study was to determine the association between individual patient sociodemographic characteristics and waiting times to physician assessment in EDs in a Swedish health care region.

## 2 Methods

This study adheres to the STROBE statement for reporting observational studies.^18^

### 2.1 Study design and setting

All EDs in Sweden are publicly funded through a decentralized single-payer system and open to any patient seeking care.^19^ We conducted an observational cohort study including all adult patients visiting the 7 EDs in the Stockholm region, Sweden, during the years 2012 to 2016, with a total of 884,228 unique patients and 1,744,301 visits. Data were collected from 1 University Hospital with 2 separate ED sites, 4 public teaching hospitals, and 1 privately operated, publicly funded hospital. The patient characteristics were those of a mixed urban, suburban, and rural area in terms of variation in socioeconomy, age, sex, and educational level. Patients were triaged utilizing the rapid emergency triage and treatment system (RETTS) with patient priority 1 to 5. RETTS is a triage system applied widely in Sweden, which prioritizes patients based on a combination of patient vitals, a structured patient anamnesis, and a trained nurse’s assessment of urgency.^20^

In the studied setting, physicians voluntarily assign themselves to patients. This decision is primarily guided by triage priority and arrival time, following a first-come, first-served order within each priority level. The triage protocol (RETTS) is based on chief complaint and vital parameters and is usually performed by a nurse and an assistant nurse.

However, the process is not strictly protocol-driven and may be influenced by other factors, eg, informal communication and interactions between physicians, nurses, and patients, which could introduce bias in how patients are selected relative to their clinical needs.

### 2.2 Selection of participants

Each visit made by patients aged 18 years or older to a general ED in Stockholm between January 1, 2012 and December 31, 2016 was included. Patients triaged as priority 1 (resuscitation) were excluded, as they follow a separate process with no measurable waiting time to a physician. Priority 5 patients were also excluded, as this category includes individuals not in need of ED care, such as patients who were deceased on arrival, those seeking administrative services, or those requiring basic care that could be managed in a nonurgent setting. Visits with missing data in the outcome or in any of the exposure or adjustment variables were excluded case-wise.

### 2.3 Exposures

The studied exposures were age, sex, educational level, and region of birth. Patients were categorized into the following age groups: 18-39 years, 40-59 years, 60-79 years, and ≥80 years. Sex was dichotomized into male and female, defined as having a male or female personal identity number for Swedish residents, and identifying as a male or female for non-residents. Patients’ educational level was categorized into 3 groups: less than 10 years, 10-12 years, and more than 12 years of formal education. Patient region of birth was classified into 4 categories: Sweden, a Nordic country, any of the current 27 EU countries, or other countries.

Patient name, age, and sex were known to physicians before seeing or signing up for the patient. Educational level and region of birth were not known specifically beforehand but might in many cases have been suggested by information on patient name, age, and sex. Patient’s spoken language(s) were not registered, and the potential ordering of interpreter service did not in itself prolong waiting time to the physician, as such services were ordered once a physician was assigned.

### 2.4 Covariates and descriptive data

Information was entered into the database from electronic health records. Chief complaint categories were selected based on those that had the highest number of deaths within 30 days. This resulted in abdominal pain, chest pain, dyspnea, peripheral edema, head injury, arrhythmia, malaise, fever, hip injury, neurologic deficit/stroke, or into “other complaints.” First assigned triage priority according to RETTS was used, of which priorities 2, 3, and 4 were included in the study. Patients were categorized as admitted or discharged. The definition of crowding level used was the hourly number of patients present in the ED in relation to the expected number of patients in the ED at that time, according to the method developed by Af Ugglas et al.^5^ ED crowding was categorized into <75%, 75%-95%, and 95%-100% where 100% represents the worst crowding level measured during the study period. Patients were categorized by the shift and date when they registered at the front desk. ED shifts were defined as day shift (07:00 am-3:00 pm), evening shift (3:00 pm-11:00 pm), and night shift (11:00 pm -07:00 am). Weekends were defined as Saturdays, Sundays, and public Swedish holidays and separated from all other days, which were defined as weekdays. Night shifts were categorized by the date preceding the shift. Hospitals were separated into their respective physical ED sites, resulting in the 7 different sites listed in Table 1.

**Table 1:** Included ED visits, exposures, and variables adjusted for in the statistical analyses.

Region of birth	Sweden		Nordics		EU27		Others		Total	
	n	%	n	%	n	%	n	%	n	%
Total	1,295,523	40.4	88,121	2.7	77,901	2.4	282,756	16.2	1,744,301	100.0
Sex										
Male	616,849	47.6	33,387	37.9	36,728	47.1	137,817	48.7	824,781	47.3
Female	678,674	52.4	54,734	62.1	41,173	52.9	144,939	51.3	919,520	52.7
Age group										
18-39 y	352,577	27.2	4000	4.5	17,745	22.8	108,275	38.3	482,597	27.7
40-59 y	315,872	24.4	20,722	23.5	18,633	23.9	118,476	41.9	473,703	27.2
60-79 y	394,938	30.5	42,450	48.2	25,788	33.1	47,613	16.8	510,789	29.3
≥80 y	232,136	17.9	20,949	23.8	15,735	20.2	8392	3.0	277,212	15.9
Educational level										
<10 y	314,074	24.2	31,901	36.2	16,162	20.7	89,722	31.7	451,859	25.9
10-12 y	571,007	44.1	37,685	42.8	32,853	42.2	100,985	35.7	742,530	42.6
>12 y	410,442	31.7	18,535	21.0	28,886	37.1	92,049	32.6	549,912	31.5
Triage priority										
2	201,200	15.5	15,676	17.8	11,717	15.0	34,116	12.1	262,709	15.1
3	582,909	45.0	43,253	49.1	35,100	45.1	118,928	42.1	780,190	44.7
4	511,414	39.5	29,192	33.1	31,084	39.9	129,712	45.9	701,402	40.2
Chief complaint										
Abdominal pain	161,275	12.4	9119	10.3	9500	12.2	46,725	16.5	226,619	13.0
Chest pain	108,020	8.3	9228	10.5	8328	10.7	34,575	12.2	160,151	9.2
Dyspnea	85,348	6.6	7244	8.2	5501	7.1	15,864	5.6	113,957	6.5
Peripheral edema	73,820	5.7	5486	6.2	4230	5.4	10,349	3.7	93,885	5.4
Head injury	43,622	3.4	3228	3.7	2229	2.9	6060	2.1	55,139	3.2
Arrythmia	42,858	3.3	3,042	3.5	2,343	3.0	4,504	1.6	52,747	3.0
Malaise	34,968	2.7	2932	3.3	2253	2.9	6665	2.4	46,818	2.7
Fever	35,574	2.7	2,221	2.5	1,980	2.5	6,930	2.5	46,705	2.7
Hip injury	28,100	2.2	2315	2.6	1508	1.9	1581	0.6	33,504	1.9
Neurologic deficit, stroke	20,916	1.6	1692	1.9	1204	1.5	2879	1.0	26,691	1.5
Other	661,022	51.0	41,614	47.2	38,825	49.8	146,624	51.9	888,085	50.9
Admission status										
Admitted	427,705	33.0	35,570	40.4	24,927	32.0	58,587	20.7	546,789	31.3
Discharged	867,818	67.0	52,551	59.6	52,974	68.0	224,169	79.3	1,197,512	68.7
Hospital										
St Göran	248,189	19.2	12,661	14.4	15,283	19.6	52,419	18.5	328,552	18.8
Danderyd	271,025	20.9	17,614	20.0	14,829	19.0	45,954	16.3	349,422	20.0
Huddinge	185,351	14.3	15,125	17.2	14,966	19.2	68,183	24.1	283,625	16.3
Norrtälje	80,041	6.2	5150	5.8	1677	2.2	3607	1.3	90,475	5.2
Solna	153,837	11.9	9126	10.4	10,970	14.1	44,693	15.8	218,626	12.5
Södertälje	77,290	6.0	9303	10.6	4258	5.5	26,675	9.4	117,526	6.7
Södersjukhuset	279,790	21.6	19,142	21.7	15,918	20.4	41,225	14.6	356,075	20.4
Shift										
Day (07:00 am - 3:00 pm)	657,491	50.8	47,054	53.4	40,513	52.0	130,212	46.1	875,270	50.2
Evening (3:00 pm - 11:00 pm)	476,417	36.8	31,144	35.3	28,376	36.4	110,131	38.9	646,068	37.0
Night (11:00 pm - 07:00 am)	161,615	12.5	9923	11.3	9012	11.6	42,413	15.0	222,963	12.8
Weekend										
Weekday	860,462	66.4	59,661	67.7	52,294	67.1	185,520	65.6	1,157,937	66.4
Weekend or holiday	435,061	33.6	28,460	32.3	25,607	32.9	97,236	34.4	586,364	33.6
Crowding level										
< 75 = low	971,586	75.0	65,917	74.8	58,821	75.5	211,980	75.0	1,308,304	75.0
75-95 = moderate	259,165	20.0	17,723	20.1	15,573	20.0	56,367	19.9	348,828	20.0
95-100 = high	64,772	5.0	4481	5.1	3507	4.5	14,409	5.1	87,169	5.0

%, share of visits belonging to each category; n, number of visits.

### 2.5 Outcome

The outcome was the waiting time to the first physician assessment, defined as the time from patient registration to physician assignment in the electronic health record.

### 2.6 Data sources

Arrival date/time, sex, age, chief complaint, and triage priority were acquired from electronic health care records in the participating hospitals. The personal identity number assigned to every Swedish resident was used to link information on patient visits to the patient’s region of birth and educational level retrieved from Statistics Sweden, and pseudonymized information was delivered to the researcher. The resulting database has been used in prior studies on crowding and patient mortality, and a new proxy for crowding was introduced by Ugglas in 2020,^5^ which has been usedin this study.^21^ Ethical approvals for this research were given by the Stockholm Regional Board of Ethics, Dnr 2014_1822-31, Dnr 2016_0569-32, and Dnr 2017_0364-32, and by the Swedish Ethical Review Authority, Dnr 2020-01691.

### 2.7 Analysis

A multivariable generalized linear regression model estimated using penalized quasi-likelihood was applied to assess the association between waiting time to a physician and sociodemographic exposures, adjusting for clinical and organizational confounders. The model assumed a gamma distribution with a log link. To account for repeated visits, a random intercept for patient ID was included. Results are presented as rate ratios.

The model included the following sociodemographic variables: sex, age, region of birth, and educational level. Adjustments were made for triage priority, chief complaint category, admission status, crowding level, shift type, weekend arrival, and hospital site. Interactions between the main exposure variables were also analyzed to assess combined differences between subgroups. Sensitivity analysis was performed, which also included patients with priority.

All analyses were conducted in R version 4.4.2 using the libraries dplyr, lubridate, forcats, readr, tidyr, MASS, and arsenal. A 95% CI and a significance level of 0.05 were used.

## 3 Results

### 3.1 Characteristics of the study subjects

**Figure 1:**
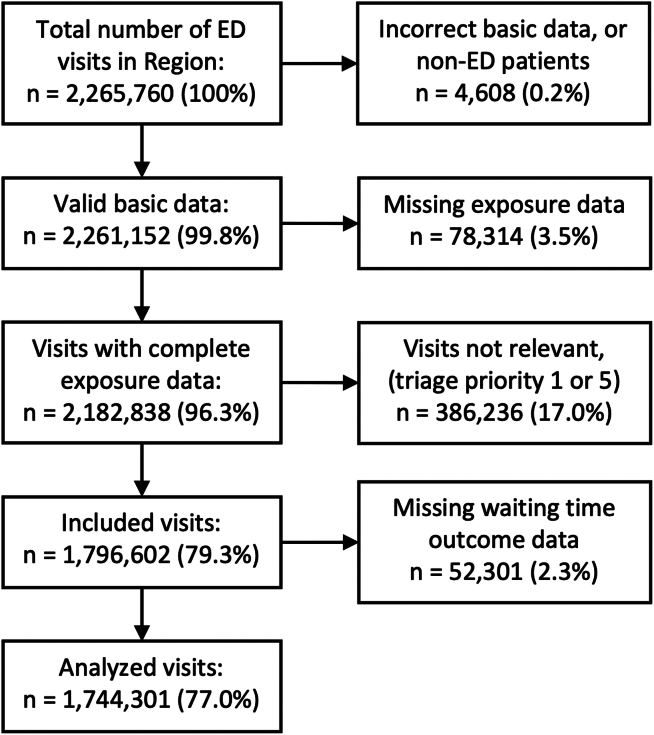
Excluded and included patient visits.

A total of 1,744,301 ED visits in the Stockholm region were analyzed. Patient visits were excluded or included according to Figure 1 below.

The distribution of patient visits by exposures and birth region is presented in Table 1. Patients born in the “others” region constituted 16.2% of included visits to the 7 EDs in the Stockholm region during the study period and were, in general, substantially younger than patients born in Sweden, the Nordics, or an EU27 country. This pattern is also reflected in the patient visit chief complaint, where, eg, hip injuries, neurologic deficit, and arrhythmia are less frequent complaints among patients from the “others” region of birth. The share of male vs female patients was similar between patients from different birth regions, as was the educational level.

### 3.2 Main results

The adjusted model included all exposures as well as triage priority, chief complaint, admission status, hospital, shift, weekend/weekday, and crowding level.

**Figure 2:**
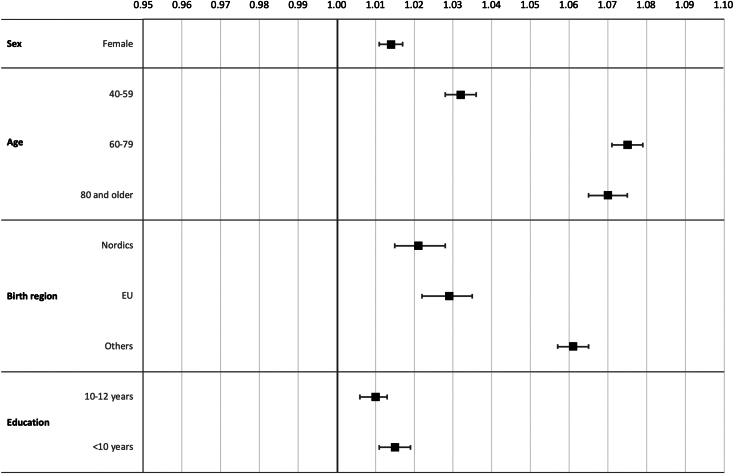
Visualization of the adjusted association between sex, age, region of birth and education and waiting time to physician presented as rate ratios with 95% CI.

The crude model intercept was 81.3 minutes (95% CI, 80.9-81.6), representing the estimated waiting time to a physician for the reference patient group: Swedish-born males aged 18 to 39 years with more than 12 years of education, who had the shortest observed waiting time. All-time differences between groups were statistically significant compared with their respective reference categories. Observed waiting time together with crude and adjusted estimates and absolute risks were presented in Table 2, and adjusted estimates were visualized in Figure 2. In the crude model, female patients had a 5% longer waiting time to physician compared to male patients. When adjusted for all other variables, 1% (95% CI, 1%-2%) remained. For elderly patients, the average waiting time to a physician was shorter compared with that of younger patients. However, when accounting for differences in triage priority and other confounding factors related to the ED visit, the adjusted estimate indicated a prolonged waiting time to physician of 3% (95% CI, 3%-4%) for patients aged 40-59 years, 8% (95% CI, 7%-8%) for patients aged 60-79 years, and 7% (95% CI, 7%-8%) for patients older than 80 years, compared with the youngest reference group.

**Table 2:** The association between sex, age, region of birth, and education and waiting time to physician, descriptive data, and multivariate estimates of rate ratios with 95% CI.

Variable	Observed waiting times, min			Estimated rate ratios in multivariable models		Absolute risk, min
	Median	IQR	Mean	Crude^a^	Adjusted^b^	Estimated increase in waiting time^c^
Sex						
Male	57	95	91	Reference	Reference	Reference
Female	60	99	94	1.05 (1.04-1.05)	1.01 (1.01-1.02)	1
Age group						
18-39 y	62	104	96	Reference	Reference	Reference
40-59 y	60	100	95	0.98 (0.97-0.98)	1.03 (1.03-1.04)	3
60-79 y	58	93	91	0.94 (0.94-0.95)	1.08 (1.07-1.08)	6
≥80 y	53	88	86	0.88 (0.88-0.89)	1.07 (1.07-1.08)	6
Region of birth						
Sweden	57	95	91	Reference	Reference	Reference
Nordics	59	94	93	1.03 (1.02-1.04)	1.02 (1.01-1.03)	2
EU	60	99	94	1.05 (1.04-1.06)	1.03 (1.02-1.03)	2
Others	65	107	100	1.09 (1.08-1.09)	1.06 (1.06-1.06)	5
Educational level						
<10 y	59	97	94	1.04 (1.03-1.04)	1.02 (1.01-1.02)	1
10-12 y	59	97	93	1.03 (1.02-1.03)	1.01 (1.01-1.01)	1
>12 y	58	96	92	Reference	Reference	Reference
Triage priority						
2	37	44	50	Not in model	Reference	
3	68	109	104	Not in model	2.01 (2.00-2.02)	
4	63	107	97	Not in model	2.30 (2.29-2.31)	
Chief complaint						
Abdominal pain	75	108	107	Not in model	1.34 (1.33-1.35)	
Chest pain	51	91	84	Not in model	1.02 (1.01-1.03)	
Dyspnea	50	85	82	Not in model	1.07 (1.06-1.08)	
Peripheral edema	67	111	103	Not in model	1.15 (1.14-1.16)	
Head injury	56	88	91	Not in model	1.17 (1.15-1.18)	
Arrythmia	47	78	78	Not in model	Reference	
Malaise	77	130	117	Not in model	1.27 (1.25-1.28)	
Fever	54	81	85	Not in model	1.16 (1.14-1.17)	
Hip injury	43	67	71	Not in model	1.05 (1.04-1.07)	
Neurologic deficiency including stroke	52	96	94	Not in model	1.07 (1.06-1.09)	
Other	58	96	92	Not in model	1.18 (1.17-1.18)	
Admission status						
Admitted	50	81	83	Not in model	Reference	
Discharged	63	104	98	Not in model	1.12 (1.11-1.12)	
Hospital						
St Göran	28	48	47	Not in model	Reference	
Danderyd	62	93	95	Not in model	2.30 (2.29-2.31)	
Huddinge	61	101	96	Not in model	2.27 (2.26-2.28)	
Norrtälje	41	61	60	Not in model	1.38 (1.37-1.39)	
Solna	70	98	98	Not in model	2.41 (2.40-2.43)	
Södertälje	58	89	84	Not in model	1.87 (1.85-1.88)	
Södersjukhuset	97	145	138	Not in model	3.32 (3.31-3.34)	
Shift						
Day (07:00 am - 3:00 pm)	48	72	71	Not in model	Reference	
Evening (3:00 pm - 11:00 pm)	76	136	119	Not in model	1.62 (1.61-1.62)	
Night (11:00 pm - 07:00 am)	67	109	102	Not in model	1.40 (1.39-1.40)	
Weekend						
Weekday	55	90	87	Not in model	Reference	
Weekend or holiday	66	112	104	Not in model	1.11 (1.11-1.12)	
Crowding level						
<75 = low	54	88	86	Not in model	Reference	
75-95 = moderate	74	121	112	Not in model	1.30 (1.29-1.30)	
95-100 = high	84	134	122	Not in model	1.47 (1.46-1.48)	

a Crude model includes sex, age, region of birth, and education level.

b Adjusted model additionally includes triage priority, chief complaint, admission status, hospital, shift type, weekend arrival, and crowding level.

c Compared with the mean waiting time (crude intercept = 81.3 minutes, calculated using estimates with 3 decimals).

The patient’s region of birth was associated with a significant increase in waiting time to the physician. There was a dose-response relationship pattern for both unadjusted and adjusted associations with waiting times to physicians, increasing progressively with greater distance of patients’ country of birth from Sweden. When adjusted for confounders, patients born in the Nordic countries were waiting 2% (95% CI, 1%-3%) longer to physician, patients born in the EU, but outside the Nordic countries were waiting 3% (95% CI: 2%-3%), and patients born outside of the EU were waiting 6% (95% CI, 6%-6%) longer compared with a Swedish-born ED patient. The educational level showed a small but significant association with waiting time to physician, with patients who had <10 years of education waiting 2% (95% CI, 1%-2%) longer and patients with 10-12 years of education waiting 1% (95% CI, 1%-1%) longer when compared with patients who had >12 years of education. Thus, also for educational level, patient waiting times to a physician displayed a dose-response relationship.

The sensitivity analysis, which also included patient visits with triage priority 5, yielded similar results as the main analysis. Interaction analysis revealed that the association between exposures and waiting time to a physician varied across patient subgroups. The estimates for each interaction term are presented in Supplementary Appendix 1 and can be used to estimate differences between subgroups. The example described in the footnote show that women aged >80 years, born outside the EU, with <10 years of education were estimated to have a 16% longer waiting time to physician assessment compared with Swedish-born men aged 18–39 years with >12 years of education, after adjusting for triage priority, chief complaint category, admission status, hospital, crowding level, and time of day and day of the week.

## 4 Discussion

This first Nordic multicenter cohort study of all 1.7 million unselected ED visits in the Stockholm region found small but significant dose-response associations between several sociodemographic characteristics and waiting time to a physician. Women, older patients, non-EU citizens, and patients with lower educational levels waited longer to be seen.

### 4.1 Sex

Women experienced 1% to 2% longer adjusted waiting times. Unadjusted differences were larger, suggesting sex-based variation in clinical factors and ED context. The reasons for these sex-based differences remain unclear, although similar patterns have been observed elsewhere.^4^^,^^17^

### 4.2 Age

After adjustment, patients over 40 waited 3% to 8% longer than younger patients. In unadjusted models, older patients appeared to wait 2% to 12% less, likely primarily due to higher triage priority. This finding is different from that of Freund et al,^16^ who found longer waits for older patients in a Paris hospital setting, also after adjusting for triage. Mohsin et al^4^ observed that patients aged 15 to 44 years had longer waits in a Sydney hospital ED, but their study lacked adjustment for hospital or temporal factors. As frailty and risk of adverse outcomes increase with age,^22^ longer waits may pose a higher clinical risk. We have previously shown that age improves the prediction of adverse outcomes and mortality also within triage levels.^15^^,^^21^ Thus, shorter adjusted waiting times for elderly patients would be consistent with medical safety in the ED.

### 4.3 Region of birth

Patients born outside Sweden experienced a 1% to 6% longer adjusted waiting time to see a physician, with a gradient linked to geographic distance from Sweden, suggesting a role for cultural or linguistic factors similar to that found in other studies.^3^^,^^4^^,^^9^, ^10^, ^11^, ^12^, ^13^^,^^23^ Nordic-born patients were grouped together due to social and linguistic similarities; EU27 citizens formed a second category. The “other” group was highly heterogeneous, potentially masking subgroup-specific effects.

### 4.4 Education

Lower education level was associated with slightly longer waits, showing a dose-response relationship. Although this study cannot determine causality, the findings are consistent with the hypothesis that socioeconomic status may contribute to differences in care inequity.^4^^,^^23^

### 4.5 Strengths and limitations

This multicenter study’s strengths lie in its large data set covering 5 years and all hospital sites in a region of 2.3 million residents, and in the quality of the data set. This allowed for adjustments for both clinical factors (triage, complaint, and admission) and ED context (hospital, shift, weekday, and crowding), all of which strongly influence waiting time in this and in previous studies, for a large, unsorted ED population.^17^^,^^24^

Although the impact of individual factors on waiting time may seem modest, the combined effect of 16 percent or a 10-minute difference between the most and least advantaged groups represents a meaningful indicator of inequity in the ED. Even modest effects can translate into substantial public health impact when a large population is exposed, as with the high volume of ED visits over time. The combined effect may increase the risk of poorer clinical outcomes for disadvantaged patients.

Region of birth served as a proxy for cultural and ethnic background. Although it lacks detail on factors like integration or time since immigration, such limitations likely bias findings toward underestimation.

We lacked data on comorbidities, which could confound results. However, adjustments for triage, chief complaint, and admission likely mitigated this bias to a large degree.

Six percent of visits were excluded due to missing data and were excluded case-wise. The low number and heterogeneous character of excluded cases likely reduce systematic bias due to missing data.

As with all observational research, residual confounding is impossible to rule out. Nonetheless, we adjusted for a broader range of potential biases than previously done and have shown that these adjustments do affect the resulting waiting times.

These results should generalize to urban and semiurban EDs in high-functioning health care systems but may vary across different triage protocols and sociodemographic settings.

### 4.6 Suggested research steps

The observed differences in waiting time suggest potential inequities rooted in the health care system, triage processes, working methods, staff bias, or other areas. As noted by Hwang et al,^3^ understanding the underlying mechanisms is key to designing interventions that promote more equitable care. Future research should focus on age and birth region as identified in this study.

This large multicenter study of all adult emergency visits in the Stockholm region over 5 years found that waiting time to the physician was independently associated with age, sex, education, and birth region, even after adjusting for clinical factors and ED context. Age above 60 years and being born outside Europe were the strongest predictors of longer waits. These findings suggest disparities not explained by medical need. As longer waits are linked to worse outcomes, further research is needed to understand mechanisms in systems, processes, working methods, and other potential sources of bias to reduce inequities in emergency care.

## Author Contributions

Björn af Ugglas and Gustav Malmer contributed to the study conception and design. Björn af Ugglas and Gustav Malmer collected and curated the data. Björn af Ugglas and Gustav Malmer performed the statistical analyses. Anna Fällman and Gustav Malmer drafted the manuscript and made preliminary interpretations. Gustav Malmer, Richard Åhlberg, Per Svensson, Eli Westerlund, and Björn af Ugglas revised the manuscript for intellectual content. All authors interpreted the data and results and approved the final version of the manuscript.

## Funding and Support

By *JACEP Open* policy, all authors are required to disclose any and all commercial, financial, and other relationships in any way related to the subject of this article as per ICMJE conflict of interest guidelines (see www.icmje.org). The authors have stated that no such relationships exist.

## Declaration of Generative AI and AI-Assisted Technologies in the Writing Process

During the preparation of this work the authors used ChatGPT-4o in order to improve language and text flow. After using this tool, the authors reviewed and edited the content as needed and take full responsibility for the content of the publication.

## Conflict of Interest

All authors have affirmed they have no conflicts of interest to declare.
