# Supplementary material for: Individual Patient Sociodemographic Characteristics Are Associated With Waiting Time to Physician Assessment in Seven Swedish Emergency Departments: An Observational 5-Year Cohort Study of Emergency Department Visits in the Stockholm Region
Source: J Am Coll Emerg Physicians Open. 2025 Sep 30;6(6):100250. doi: 10.1016/j.acepjo.2025.100250 (PMC12514495; doi:10.1016/j.acepjo.2025.100250)
Supplement: Supplementary Table 1-3 [file mmc1.docx]

# Supplementary Appendix 1

The association between sex, age, region of birth and education and waiting time to physician, multivariate adjusted estimates of rate ratios including interactions with 95 % CI

| **Variable** |  | **Estimate with 95%CI** |
| --- | --- | --- |
|  | (Intercept) |  |
| **Sex** | Male | Reference |
|  | Female | 1.01 (1.00–1.02)* |
| **Age** | 18-39 | Reference |
|  | 40-59 | 1.01 (1.00–1.02) |
|  | 60-79 | 1.06 (1.06–1.07) |
|  | 80 or older | 1.07 (1.06–1.09)* |
| **Birth region** | Sweden | Reference |
|  | Nordics | 1.06 (1.02–1.09) |
|  | EU | 1.04 (1.02–1.06) |
|  | Others | 1.06 (1.05–1.07)* |
| **Education** | <10 years | 0.98 (0.97–0.99)* |
|  | 10-12 years | 0.99 (0.99–1.00) |
|  | >12 years | Reference |
| **Triage priority** | 2 | Reference |
|  | 3 | 2.01 (2.00–2.02) |
|  | 4 | 2.30 (2.29–2.31) |
| **Chief complaint** | Abdominal pain | 1.34 (1.33–1.35) |
|  | Chest pain | 1.02 (1.01–1.03) |
|  | Dyspnea | 1.08 (1.07–1.09) |
|  | Peripheral edema | 1.15 (1.14–1.16) |
|  | Head injury | 1.17 (1.15-1.18) |
|  | Arrythmia | Reference |
|  | Malaise | 1.27 (1.25–1.28) |
|  | Fever | 1.16 (1.14–1.17) |
|  | Hip injury | 1.05 (1.04-1.07) |
|  | Neurological deficit, stroke | 1.07 (1.06–1.09) |
|  | Other | 1.18 (1.17–1.18) |
| **Admission status** | Admitted | Reference |
|  | Discharged | 1.12 (1.12–1.12) |
| **Hospital** | S:t Göran | Reference |
|  | Danderyd | 2.30 (2.29–2.31) |
|  | Huddinge | 2.27 (2.26–2.28) |
|  | Norrtälje | 1.38 (1.37–1.39) |
|  | Solna | 2.41 (2.40–2.43) |
|  | Södertälje | 1.87 (1.85–1.88) |
|  | Södersjukhuset | 3.32 (3.31–3.34) |

Supplementary Appendix 1 continued: The association between sex, age, region of birth and education and waiting time to physician, multivariate adjusted estimates of rate ratios including interactions with 95 % CI

| **Variable** |  | **Estimate with 95%CI** |
| --- | --- | --- |
| **Type of shift** | Day (07:00-15:00) | Reference |
|  | Evening (15:00-23:00) | 1.62 (1.61–1.62) |
|  | Night (23:00-07:00) | 1.40 (1.39–1.40) |
| **Type of day** | Weekday | Reference |
|  | Weekend or holiday | 1.12 (1.11–1.12) |
| **Crowding** | < 75 = low | Reference |
|  | 75-95 = moderate | 1.30 (1.29–1.30) |
|  | 95-100 = high | 1.47 (1.46–1.48) |
| **Sex & Age** | sexFemale:age_group40-59 | 1.00 (1.00–1.01) |
|  | sexFemale:age_group60-79 | 1.00 (0.99–1.01) |
|  | sexFemale:age_group80 or older | 0.98 (0.97–0.98)* |
| **Sex & Birth region** | sexFemale:birth_region_groupOther | 0.99 (0.98–0.99)* |
|  | sexFemale:birth_region_groupEU27 | 1.00 (0.99–1.01) |
|  | sexFemale:birth_region_groupNordics | 0.98 (0.97–0.99) |
| **Sex & Education** | sexFemale:education_group<10 years | 1.03 (1.02–1.03)* |
|  | sexFemale:education_group10-12 years | 1.01 (1.01–1.02) |
| **Age & Birth region** | age_group40-59:birth_region_groupOther | 1.01 (1.01–1.02) |
|  | age_group60-79:birth_region_groupOther | 1.00 (0.99–1.01) |
|  | age_group80 or older:birth_region_groupOther | 1.01 (0.99–1.03)* |
|  | age_group40-59:birth_region_groupEU27 | 1.00 (0.98–1.02) |
|  | age_group60-79:birth_region_groupEU27 | 0.98 (0.96–1.00) |
|  | age_group80 or older:birth_region_groupEU27 | 0.97 (0.95–0.99) |
|  | age_group40-59:birth_region_groupNordics | 1.00 (0.97–1.03) |
|  | age_group60-79:birth_region_groupNordics | 0.99 (0.96–1.02) |
|  | age_group80 or older:birth_region_groupNordics | 1.01 (0.98–1.05) |
| **Age & Education** | age_group40-59:education_group<10 years | 1.03 (1.02–1.04) |
|  | age_group60-79:education_group<10 years | 1.04 (1.03–1.05) |
|  | age_group80 or older:education_group<10 years | 1.02 (1.01–1.03)* |
|  | age_group40-59:education_group10-12 years | 1.02 (1.01–1.03) |
|  | age_group60-79:education_group10-12 years | 1.02 (1.01–1.03) |
|  | age_group80 or older:education_group10-12 years | 1.02 (1.01–1.03) |
| **Birth region & Education** | birth_region_groupOther:education_group<10 years | 1.01 (1.00–1.02)* |
|  | birth_region_groupEU27:education_group<10 years | 1.00 (0.98–1.02) |
|  | birth_region_groupNordics:education_group<10 years | 0.97 (0.95–0.98) |
|  | birth_region_groupOther:education_group10-12 years | 1.00 (0.99–1.01) |
|  | birth_region_groupEU27:education_group10-12 years | 1.00 (0.99–1.02) |
|  | birth_region_groupNordics:education_group10-12 years | 0.98 (0.97–1.00) |
| * included in combined estimate for female, age >80, Born outside EU with <10 years education | | |
| 1.01*1.07*1.06*0.98*0.98*0.99*1.03*1.01*1.02*1.01 = 1.16 (calculation performed with 3 decimals) | | |
